# Supplementary material for: QTc Interval Reference Values and Their (Non)-Maturational Factors in Neonates and Infants: A Systematic Review
Source: Children (Basel). 2022 Nov 18;9(11):1771. doi: 10.3390/children9111771 (PMC9688724; doi:10.3390/children9111771)
Supplement: Supplementary file 1 [file children-09-01771-s001.zip › children-2002455-supplementary.pdf]

Supplemental Materials

Table S1. Search terms in Pubmed, Embase, Web of Science and Cochrane Library.

Table S2. Search entries in Pubmed, Embase, Web of Science and Cochrane Library.

Table S3. Quality assessment of randomized and non-randomized controlled trials using the RoB 2 [9] and ROBINS-I tool [10].

Table S4. Quality assessment of case-control studies organized by date using the Newcastle Ottawa Scale (NOS) tool containing four domains of selection, one domain of comparability and three domains of exposure [11].

Table S5. Quality assessment of cohort studies organized by date using the Newcastle Ottawa Scale (NOS) tool containing four domains of selection, one domain of comparability and three domains of exposure [11].

Table S6. Quality assessment of cross-sectional studies organized by date using the Newcastle Ottawa Scale (NOS) tool containing four domains of selection, one domain of comparability and two domains of exposure [11].

Table S7: Data extraction of 18 articles concerning maturational effects - organized by outcome.

Table S8. Data extraction of 9 articles concerning covariates influencing QTc - organized by covariate.

Table S9. Data extraction of 23 articles concerning medicines influencing QTc - organized by medicine.

Table S1. Search terms in Pubmed, Embase, Web of Science and Cochrane Library.

**PRETERM - TERM - NEWBORN - INFANT**	
**Pubmed**	"Premature Birth"[Mesh] OR Prematur*[tiab] OR Preterm[tiab] OR "pre term"[tiab] OR "Term Birth"[Mesh] OR Term Birth*[tiab] OR Fullterm Birth*[tiab] OR Neonat*[tiab] OR Newborn*[tiab]; OR "Infant"[Mesh] OR infan*[tiab] NOT "Review" [Publication Type]
**Embase**	'Prematurity'/exp OR 'Prematur*':ti,ab,kw OR 'Pre term':ti,ab,kw OR 'Preterm':ti,ab,kw; OR 'Term birth'/exp OR 'Term birth*':ti,ab,kw OR 'Fullterm birth*':ti,ab,kw; OR 'Neonat*':ti,ab,kw OR 'Newborn*':ti,ab,kw; OR 'Infant'/exp OR 'Infan*':ti,ab,kw
**Web of Science**	TS=("Prematur*" OR "Preterm" OR "pre term"); OR TS=("Term Birth*" OR "Fullterm Birth*"); OR TS=("Neonat*" OR "Newborn*"); OR TS=("Infan*")
**Cochrane**	#1: ([mh "Premature Birth"] OR [mh "Term Birth"] OR [mh "Infant"])<br>#2: (Prematur* OR Preterm OR "pre term" OR (Term NEXT Birth*) OR (Fullterm NEXT Birth*) OR Neonat* OR Infan*):ti,ab,kw<br>#3: #1 OR #2
**QTc - QT - ELECTROCARDIOGRAPHY**	
**Pubmed**	"Electrocardiography"[Mesh:NoExp] OR Electrocardiogra*[tiab] OR EKG[tiab] OR ECG[tiab] OR QT[tiab] OR QTc[tiab] ; NOT "Review" [Publication Type]
**Embase**	'Electrocardiography'/de OR 'Fetus electrocardiography'/exp OR 'Electrocardiogra*':ti,ab,kw; OR 'EKG':ti,ab,kw OR 'ECG':ti,ab,kw; OR 'QT':ti,ab,kw OR 'QTc':ti,ab,kw
**Web of Science**	TS=("electrocardiogra*"); OR TS=("EKG" OR "ECG"); OR TS=("QTc" OR "QT")
**Cochrane**	#4: ([mh "Electrocardiography"])<br>#5: (Electrocardiogra* OR ECG OR EKG OR QT OR QTc):ti,ab,kw<br>#6: #4 OR #5; #7: #3 AND #6

Table S2. Search entries in Pubmed, Embase, Web of Science and Cochrane Library.

**Pubmed**	("Premature Birth"[Mesh] OR Prematur*[tiab] OR Preterm[tiab] OR Term Birth[Mesh] OR Term Birth*[tiab] OR Fullterm Birth*[tiab] OR Neonat*[tiab] OR Newborn*[tiab] OR "Infant"[Mesh] OR infant*[tiab]) AND ("Electrocardiography"[Mesh:NoExp] OR Electrocardiogra*[tiab] OR EKG[tiab] OR ECG[tiab] OR QT[tiab] OR QTc[tiab]) NOT "Review" [Publication Type])
**Embase**	(‘Prematurity’/exp OR ‘Prematur*’:ti,ab,kw OR ‘Pre term’:ti,ab,kw OR ‘Preterm’:ti,ab,kw OR 'Term birth'/exp OR 'Term birth*':ti,ab,kw OR ‘Fullterm birth*’:ti,ab,kw OR ‘Neonat*’:ti,ab,kw OR ‘Newborn*’:ti,ab,kw OR 'Infant'/exp OR 'Infant*':ti,ab,kw)<br>AND (‘Electrocardiography'/de OR 'Fetus electrocardiography'/exp OR ‘Electrocardiogra*’:ti,ab,kw OR ‘EKG’:ti,ab,kw OR ‘ECG’:ti,ab,kw OR ‘QT’:ti,ab,kw OR ‘QTc’:ti,ab,kw) NOT ‘conference abstract’:it NOT ‘review’:it
**Web of Science**	(TS=(“Prematur*” OR “Preterm” OR “pre term”) OR TS=(“Term Birth*” OR “Fullterm Birth*”) OR TS=(“Neonat*” OR “Newborn*”) OR TS=(“Infan*”)) AND (TS=(“electrocardiogra*”) OR TS=(“EKG” OR “ECG”) OR TS=(“QTc” OR “QT”)
**Cochrane**	#1: ([mh “Premature Birth”] OR [mh “Term Birth”] OR [mh “Newborn”] OR [mh “Infant”])<br>#2: (“Prematur*” OR “Preterm” OR “Term Birth*” OR “Fullterm Birth*” OR “Neonat*” OR “Infant”):ti,ab,kw<br>#3: #1 OR #2<br>#4 = ([mh "Electrocardiography"])<br>#5 = (“Electrocardiogra*” OR ECG OR EKG OR QT OR QTc):ti,ab,kw<br>#6: #4 OR #5<br>#7: #3 AND #6

Table S3. Quality assessment of randomized and non-randomized controlled trials using the RoB 2 [9] and ROBINS-I tool [10].

	D1	D2	D3	D4	D5	D6	D7	D8	D9
Ramirez-Mayans, 2000 [43]	?	+			+	?	+	+	+
[42] Costalos, 2000			+	?	+	+	+	?	+
[57] Kohl, 2005			+		+	+	+	+	+

Domains:
D1 Bias due to confounding
D2 Bias in selection of participants into the study
D3 Randomization process
D4 Bias arising from period and carryover effects
D5 Bias due to derivations from intended interventions
D6 Bias due to missing data
D7 Bias in measurements of the outcome
D8 Bias in selection of the reported results
D9 Overall bias

Risk of bias:
+ Low risk
? Some concerns
- High risk

Table S4. Quality assessment of case-control studies organized by date using the Newcastle Ottawa Scale (NOS) tool containing four domains of selection, one domain of comparability and three domains of exposure [11].

Study		Selection				Comparability	Exposure			
Author	Year	Is the case definition adequate?	Representativeness of the cases	Selection of controls	Definition of controls	Comparability of cases and controls on the basis of the design or analysis	Ascertainment of exposure	Same method of ascertainement for cases and controls	Non-response rate	Stars
Kelly [64]	1977	*	*	*	*	**	*	*	*	**9**
Haddad, Epstein [67]	1979	*	*	*	*	*	*	*	*	**8**
Montague [65]	1984	*		*	*	**	*	*	*	**8**
Weinstein [68]	1985	*	*	*			*	*	*	**6**
Vandenplas [55]	2000	n/a	n/a	n/a	n/a	**	*	*	n/a	**4**
Benatar [53]	2001	*	*	*	*	*		*	*	**7**
Corvaglia [56]	2004	*	*	*	*	**	*	*	*	**9**
Dubnov-Raz [7]	2008	*	*	*	*	*		*	*	**8**
Parikh [78]	2011	*	*	*	*	**	*	*	*	**9**

Table S5. Quality assessment of cohort studies organized by date using the Newcastle Ottawa Scale (NOS) tool containing four domains of selection, one domain of comparability and three domains of exposure [11].

Study		Selection				Comparability	Outcome			
Author	Year	Representativeness of exposed cohort	Selection of the non exposed cohort	Ascertainment of the exposure	Demonstration that outcome of interest was not present at start of study	Comparability of cohorts on the basis of the design or analysis	Assessment of outcome	Was follow up long enough for outcomes to occur?	Adequacy of follow up for cohorts	Stars
Hubscher [19]	1961		n/a	*	*	n/a	*	*	*	**5**
Wenger [15]	1961		n/a	*	*	n/a	*	*	*	**5**
Walsh [20]	1963	*	n/a	*	*	n/a	*	*	*	**6**
Walsh [32]	1975	*	n/a	*	*	*		n/a	n/a	**4**
Giacocia [37]	1978	*	*	*	*	**	*	*	*	**8**
Haddad, Krongrad [38]	1979	*	n/a	*	*	n/a		*	*	**5**
Schwartz [25]	1982	*	n/a	*	*	n/a	*	*	*	**6**
Southall [66]	1986	*	*	*	*	**	*	*	*	**9**
Thomaidis [23]	1988	*	n/a	*	*	n/a	*	*	*	**6**
Schaffer [21]	1991	*	n/a	*	*	n/a	*	*	*	**6**
Stramba-Badiale [30]	1955	*	n/a	*	*	n/a			*	**4**
Bernardini [44]	1997	*	n/a	*	*	*	*	*	*	**7**
Khogphatthanayothin [45]	1998	*	n/a	*		n/a	*	*	*	**5**
Schwartz [69]	1998	*	*	*	*	**	*	*	*	**9**
Cools [52]	2001		n/a	*	*	n/a	*		*	**4**

Dubin [50]	2001	*	n/a	*	*	n/a	*	*	*	**6**
Maillard [63]	2001	*	n/a	*	*	n/a	*	*	*	**6**
Semama [46]	2001	*	n/a	*	*	*	*	*	*	**7**
Zamora [47]	2001	*	n/a	*	*	n/a	*		*	**5**
Benatar, Cools [54]	2002		n/a	*	*	n/a	*		*	**4**
Benatar, Ramet [41]	2002	*	n/a	*	*	n/a		*	*	**5**
Chhina [49]	2002	*	n/a	*	*	n/a	*	*	*	**6**
Ariagno [39]	2003	*	n/a	*	*	n/a		*	*	**5**
Cools [51]	2003		n/a	*	*	n/a	*		*	**4**
Zamora [48]	2004	*	n/a	*	*	n/a	*		*	**5**
Berul [58]	2006	*	n/a	*	*	n/a	*	*	*	**6**
Horan [34]	2007		n/a	*	*	*	*	*	*	**6**
Miyata [62]	2007		n/a	*	*	n/a	*		*	**4**
Djeddi [59]	2008	*	n/a	*	*	n/a	*		*	**5**
Marti-Almor [24]	2008	*	n/a	*	*	n/a			*	**4**
Millat [70]	2009	*	*	*	*	*	*	*	*	**8**
Günlemez [60]	2010	*	n/a	*	*	n/a	*	*	*	**6**
Krasemann [31]	2010	*	n/a	*	*	n/a		*	*	**5**
Makarov [16]	2010	*	n/a	*	*	n/a	*	*	*	**6**
Vieira [61]	2012	*	n/a	*	*	n/a	*	*	*	**6**
Ulrich [22]	2014	*	n/a	*	*	n/a		*	*	**5**
Shabestari [36]	2019	*	*	*	*	**	*	*	*	**9**
Friedman [79]	2020		n/a	*	*	n/a		*	*	**4**

Marcellino [72]	2021	*	n/a	*	*	n/a	*	*	*	**6**
Paerregaard [18]	2021	*	n/a	*	*	n/a	*	*	*	**6**

Table S6. Quality assessment of cross-sectional studies organized by date using the Newcastle Ottawa Scale (NOS) tool containing four domains of selection, one domain of comparability and two domains of exposure [11].

Study		Selection				Comparability	Exposure		
Author	Year	Representativeness of the sample	Sample size	Non-respondents	Ascertainment of the exposure	The subjects in different outcome groups are comparable, based on the study design or analysis	Assessment of the outcome	Statistical test	**Stars**
Walsh [17]	1964	*	*	*	**		**	*	**8**
Emmanouillides [33]	1965	*	*		**	n/a			**4**
Rijnbeek [26]	2001	*	*	*	**	n/a	**	*	**8**
Semizel [29]	2008	*	*		**	n/a	**	*	**7**
Yoshinaga [28]	2013	*	*		**	n/a	**	*	**7**
Uygur [27]	2019	*	*		**	n/a	*	*	**6**

Table S7: Data extraction of 18 articles concerning maturational effects - organized by outcome.

<table>
<tr><th>Author
(year)</th><th>Title</th><th>Patients
n*</th><th>Study population
characteristics</th><th>QT or QTc-interval
Key findings</th></tr>
<tr>
<td>Walsh
(1963) [20]</td>
<td>Electrocardiographic intervals during the first week of life</td>
<td>68 full term infants</td>
<td>Divided based on age at first examination
(1) 15-30 min old
(2) 35-60 min old
(3) 65 min – 4h old
(4) 4 – 18h old</td>
<td>QTc interval: significant decrease in maximum values during the first week of life
Distribution of cases according to QTc during the first week of life
<table>
<tr><td rowspan="2">Day</td><td colspan="4">QTc interval (ms) (lead II)</td></tr>
<tr><td>340-420</td><td>430-480</td><td>490-540</td><td>SD</td></tr>
<tr><td>1</td><td>20</td><td>33</td><td>13</td><td>0.045</td></tr>
<tr><td>2</td><td>14</td><td>42</td><td>11</td><td>0.038</td></tr>
<tr><td>3</td><td>16</td><td>48</td><td>3</td><td>0.033</td></tr>
<tr><td>5/6</td><td>29</td><td>38</td><td>1</td><td>0.025</td></tr>
</table>
</td>
</tr>
<tr>
<td>Makarov
(2010) [16]</td>
<td>QT dynamicity, microvolt T wave alternans, and heart rate variability during 24-hour ambulatory electrocardiogram monitoring in the healthy newborn of the first day of life</td>
<td>20 healthy newborns
16 M
4 F</td>
<td>Inclusion criteria
(1) Normal pregnancy
(2) Healthy mother: 18-28 years
(3) GA: 38-40 weeks
(4) Body mass ≥ 2700 g
(5) Apgar score ≥ 8
(6) absence of cardiovascular pathology
(7) 12 lead ECG at rest</td>
<td>QTc interval on the first day of life

First day of life: increased slope QT/RR (steep slope) compared with older children
A steep slope = sign of hyperadaptation of QT to HR
A flat slope = sign of hypoadaptation of QT to HR

Mean values ± SD (ms)
<table>
<tr><td>Age</td><td>Day 1</td><td>Day 2</td><td>Day 4</td><td>D 1-4 (average)</td></tr>
<tr><td>mean QT interval at minimal HR</td><td>347 ± 25</td><td>343 ± 22</td><td>328 ± 20</td><td>340 ± 34</td></tr>
<tr><td>Longest QT interval</td><td>356 ± 21</td><td>351 ± 16</td><td>336 ± 19</td><td>342 ± 38</td></tr>
</table>
</td>
</tr>
</table>

<table>
<tr>
<td></td>
<td></td>
<td></td>
<td></td>
<td>
<table>
<tr><td>QTcB (Bazett)</td><td>434 ± 7</td><td>458 ± 12*</td><td>438 ± 7</td><td>440 ± 13</td></tr>
<tr><td>QTcF (Fridericia)</td><td>382 ± 4</td><td>393 ± 8*</td><td>385 ± 5</td><td>388 ± 12</td></tr>
<tr><td>QTpc (corrected QT peak interval by Bazett)</td><td>302 ± 11</td><td>313 ± 24</td><td>299 ± 7</td><td>306 ± 15</td></tr>
<tr><td>Slope QT/RR</td><td>0.35 ± 0.06</td><td>0.36 ± 0.03</td><td>0.44 ± 0.07*</td><td>0.25 ± 0.07</td></tr>
</table>
* p < 0.001, between max and min mean values in first second or fourth days of life
</td>
</tr>
<tr>
<td>Hubsher
(1961) [19]</td>
<td>The electrocardiogram of the premature infant</td>
<td>143 preterm infants</td>
<td>(1) 800-1300 g
(2) 1300-1800 g
(3) 1800-2300 g</td>
<td>
QTc interval prolonged on first day of life
Mean QTc (ms) ± standard deviation (in brackets)
<table>
<tr><td></td><td>Day 1</td><td>6 weeks</td><td>3 months</td></tr>
<tr><td>800-1300g</td><td>440 (± 30)</td><td>390 (± 20)</td><td>430 (± 10)</td></tr>
<tr><td>1300-1800g</td><td>420 (± 20)</td><td>380 (±20)</td><td>400 (± 20)</td></tr>
<tr><td>1800-2300g</td><td>440 (± 70)</td><td>400 (± 10)</td><td>400 (± 20)</td></tr>
</table>
</td>
</tr>
<tr>
<td>Wenger
(1961) [15]</td>
<td>A preliminary study of the electrocardiogram of the normal premature infant</td>
<td>7 preterm infants</td>
<td>Birth weight
< 1500 g</td>
<td>
QT interval decreases with increasing age and heart rate
Average QT interval without variability or SD given
• 0-48h = 288 ms
• 48-96h = 351 ms
• d4-d7 = 254 ms
• Week 2 = 245 ms
• Week 3 = 220 ms
• Week 4-6 = 235 ms
</td>
</tr>
<tr>
<td>Walsh
(1964) [17]</td>
<td>Comparative study of electrocardiograms of healthy premature and</td>
<td>37 preterm infants

68 full term infants</td>
<td>Each population into 2 groups based on weight
(1) < 3350 g</td>
<td>
Preterm neonates: significant shorter QT intervals than full term neonates
Mean QT (ms) with SD (in brackets)
<table>
<tr><td></td><td>< 3350 g</td><td>>3350 g</td></tr>
</table>
</td>
</tr>
</table>

<table>
<tr>
<td></td>
<td>full-term infants of similar weight</td>
<td></td>
<td>17 preterm (PT)
38 full term (FT)

(2) > 3350 g
20 preterm (PT)
30 full term (FT)</td>
<td>
<table>
<tr><td>Preterm</td><td>241 (±18.7) *</td><td>245 (±49.5) *</td></tr>
<tr><td>Full term</td><td>264 (±23.6) *</td><td>272 (±28.5) *</td></tr>
</table>
* Significant (p < 0.0005)
</td>
</tr>
<tr>
<td>Schwartz (1982) [25]</td>
<td>The QT interval throughout the first 6 months of life: a prospective study</td>
<td>4205 newborns</td>
<td>(1) normal, healthy newborns
(2) various illnesses
(3) preterm</td>
<td>Mean QTc interval (ms) with SD (in brackets)
<table>
<tr><td></td><td>Healthy newborns</td><td>Newborns with various ilnessess</td><td>Preterm newborns</td><td>Proglongation</td></tr>
<tr><td>4th day</td><td>397 (±18)
(n = 3946)</td><td>397 (±22)
(n = 141)</td><td>401 (±24)
(n = 141)</td><td>451</td></tr>
<tr><td>2nd month</td><td>409 (±15)
(n= 2418)</td><td>398 (±18)
(n = 60)</td><td>400 (±17)
(n = 51)</td><td>454</td></tr>
<tr><td>4th month</td><td>406 (±15)
(n = 351)</td><td colspan="2" rowspan="2">Not measured</td><td>451</td></tr>
<tr><td>6th month</td><td>400 (±14)
(n = 234)</td><td>442</td></tr>
</table>
n = number of patients in each group
</td>
</tr>
<tr>
<td>Thomaidis (1988) [23]</td>
<td>Comparative study of the electrocardiograms of healthy full term and premature newborns</td>
<td>421 full term and preterm newborns
(1) full term: GA 39-41 weeks
(2) preterm: GA 26-37 weeks</td>
<td>Birth weight (BW)
(1) 2501-4500 g (n= 180)
(2) 1901- 2500 g (n= 100)
(3) 1301-1900 g (n= 91)
(4) 700- 1300 g (n= 50)</td>
<td>Mean QTc values (ms) on the fifth day of life (5th and 95th percentile (in brackets))
<table>
<tr><td rowspan="2">ECG parameter</td><td rowspan="2">Full term

Group (1)
(n= 180)</td><td colspan="3">Preterm</td></tr>
<tr><td>Group (2)
(n= 100)</td><td>Group (3)
(n= 91)</td><td>Group (4)
(n= 50)</td></tr>
<tr><td>QTc interval (ms)</td><td>404 (361-442)</td><td>412 (380-467) *</td><td>414 (366- 476) *</td><td>412 (346-461)</td></tr>
</table>
n = number of patients in each group
* Statistically significant difference (p<0.05) compared to group 1
</td>
</tr>
</table>

<table>
<tr>
<td>Schaffer (1991) [21]</td>
<td>The longitudinal time course of QTc in early infancy</td>
<td>1101 newborns</td>
<td>Divided according to ethnicity
- White (W): 246
- Non-white (NW): 753

Mean birth weight: 3.200 g</td>
<td>Values at 1 week are significantly shorter ($p < 0.001$)
Transient, significant decrease in the QTc during first month of life

Mean QTc values ± SD in ms
<table>
<tr><th></th><th>Male W</th><th>Female W</th><th>Male NW</th><th>Female NW</th></tr>
<tr><td>1 day</td><td>414.72 ± 22.67</td><td>416.06 ± 20.85</td><td>415.47 ± 22</td><td>415.50 ± 20.15</td></tr>
<tr><td>1 week</td><td>403.44 ± 10.28</td><td>405.31 ± 11.88</td><td>404.11 ± 13.44</td><td>404.06 ± 11.70</td></tr>
<tr><td>1 month</td><td>416.42 ± 11.17</td><td>416.44 ± 13.34</td><td>413.46 ± 10.44</td><td>413.63 ± 11.35</td></tr>
<tr><td>2 months</td><td>419.88 ± 13.17</td><td>418.50 ± 11.79</td><td>416.05 ± 12.30</td><td>414.80 ± 11.39</td></tr>
<tr><td>3 months</td><td>417.13 ± 13.56</td><td>413.70 ± 10.14</td><td>416.76 ± 12.42</td><td>419.51 ± 14.95</td></tr>
</table>

Longitudinal time course of QTc values
<table>
<tr><th></th><th>Day 1 (0-3d)</th><th>1 week (4-10d)</th><th>1 month (11-45d)</th><th>2 months (46-75d)</th><th>3 months (76-125d)</th></tr>
<tr><td>Mean QTc (ms) ± SD</td><td>415.4 ± 21.3</td><td>404.1 ±12.1</td><td>414.2 ± 11.3</td><td>416.5 ±12.2</td><td>417.3 ±13.4</td></tr>
</table>
⟶ Significant decrease in the QTc at 1 week when compared with other ages ($p < 0.001$)</td>
</tr>
<tr>
<td>Marti-Almor (2008) [24]</td>
<td>QT interval in newborns of different ethnic origin: usefulness of neonatal ECG screening</td>
<td>1305 full term newborns between 0 and 48 hours old
51.5% M</td>
<td>Divided in 11 ethnic groups</td>
<td>Mean QTc: 417.79ms (± 28.47ms) with no significant sex and ethnical differences

If prolongation > 440 ms: 18.33% prolonged QTc interval > 440ms
17.9% Spanish
27.7% Maghrebi en Near Eastern
28.2% Indian-Pakistani
If prolongation > 97.5% percentile with 471.68ms: 4.52%
3.8% Spanish
10.9% Maghrebi and Near Eastern
No more statistical significance in Indian-Pakistani group</td>
</tr>
</table>

<table>
<tr>
<td>Stramba-badiale (1995) [30]</td>
<td>Are gender differences in QTc precent at birth?</td>
<td>33034 newborns</td>
<td>/</td>
<td>No significant effect of gender on QTc interval.
ECG recordings on the third or fourth day of life
<table>
<tr><td>ECG parameter</td><td>Overall population</td><td>Females (F)</td><td>Males (M)</td></tr>
<tr><td>Mean HR ± SD (bpm)</td><td>135 ± 20</td><td>127 ± 20 *</td><td>134 ± 20 *</td></tr>
<tr><td>Mean QT ± SD (ms)</td><td>274 ± 28</td><td>272 ± 27 *</td><td>276 ± 29 *</td></tr>
<tr><td>Mean QTc ± SD (ms)</td><td>400 ± 20</td><td>400 ± 20</td><td>401 ± 19</td></tr>
</table>
* Significant p < 0.001</td>
</tr>
<tr>
<td>Yoshinaga (2013) [28]</td>
<td>Electro-cardiographic screening of 1 month old infants for identifying prolonged QT intervals</td>
<td>4285 Japanese infants from 16 maternity institutes</td>
<td>/</td>
<td>Mean QT± SD (ms), mean HR ± SD (bpm) and mean QTc ± SD (ms) at the age of one month
<table>
<tr><td></td><td>Mean QT ± SD</td><td>Mean HR ± SD</td><td>Mean QTc ± SD</td></tr>
<tr><td>Male infants</td><td>253 ± 17 ms</td><td>160 ± 6 bpm</td><td>410 ± 19 ms</td></tr>
<tr><td>Female infants</td><td>255 ± 17 ms</td><td>158 ± 6 bpm</td><td>413 ± 19 ms</td></tr>
<tr><td>All infants</td><td>254 ± 17 ms</td><td>159 ± 16 bpm</td><td>412 ± 19 ms</td></tr>
</table>

Mean QTc of female infants is longer than male infants (p < 0.0001)
ECG screening of 1 month old infants is successful in identifying infants with prolonged QT intervals
5 infants with QTc ≥ 470 ms ⟶ 4 infants diagnosed with LQTS
QTc > 470 ms = best cut-off to screen for infants with prolonged QT intervals (PPV = 80% and NPV = 100%)
</td>
</tr>
<tr>
<td>Marcellino (2021) [72]</td>
<td>Single-centre retrospective analysis of the best timing for the QTc interval length assessment in neonates</td>
<td>3467 healthy neonates aged 14 to 30 days of life
65.3% M
43.7% F</td>
<td>After first ECG measurement: divided population in 3 groups
(1): normal QTc
(2): prolonged QTc
(3): Short QTc</td>
<td>First ECG evaluation before 30 days of life.
→ If QTc is abnormal: re-evaluation after 21 days until normalisation or transfer to a tertiary centre

Cutt-offs for QT interval

Short: < 340 ms
Normal: 340-440 ms
Prolonged borderline: 440-460 ms
Prolonged pathological: > 460 ms
</td>
</tr>
</table>

<table>
<tr><td></td><td></td><td></td><td></td><td>No correlation between QTc and BW, GA and positive family history for congenital heart disease
Females significantly longer QTc than males (p = 0.01)

Mean QTc ± SD (ms)

⟶ Female: 398 ± 29

⟶ Male: 397 ± 33

First ECG evaluation: 3467 neonates
Median age: 26 days
Median QTc = 398 ms
Prolonged 7.2% (n: 249)
Second ECG evaluation: 248 neonates (12 lost to FU)
Median age: 47 days
Median QTc: 404 ms
240 with prolonged QTc interval ⟶ 11 persistent QTc > 440 ms (4.6%)
The QTc at second ECG: significantly lower than the one measured at first ECG
Third ECG evaluation: 9 neonates (2 lost to FU)
Mean age: 67 days
Median QTc: 389 ms
1 persistent prolonged QTc (QTc 458 ms) (11.1%)</td></tr>
<tr><td>Rijnbeek (2001) [26]</td><td>New normal limits for the paediatric electrocardiogram</td><td>1912 children aged 11 days to 16 years</td><td>9 groups based on age
(1) 0-1 m (n = 44)
(2) 1-3 m (n = 138)
(3) 3-6 m (n = 182)
(4) 6-12 m (n = 235)
(5) 1-3 years
(6) 3-5 years
(7) 5-8 years
(8) 8-12 years
(9) 12-16 years</td><td>QTc remains relatively stable over the years

Median QTc (ms) (2nd percentile, 98th percentile)<table><tr><td></td><td>0-1 month</td><td>1-3 months</td><td>3-6 months</td><td>6-12 months</td></tr><tr><td>M</td><td>413 (378-448)</td><td>419 (396-458)</td><td>422 (391-453)</td><td>411 (379-499)</td></tr><tr><td>F</td><td>420 (379-462)</td><td>424 (381-454)</td><td>418 (386-448)</td><td>414 (381-446)</td></tr></table></td></tr>
</table>

<table>
<tr>
<td>Semizel
(2008) [29]</td>
<td>The effect of age and gender on the electrocardiogram in children</td>
<td>2241 healthy Turkish children between 1 day 1 and 16 years old</td>
<td>12 groups according to age and sex
(1) 0-1 d (n= 256)
(2) 1-3 d (n= 149)
(3) 3-7 d (n= 92)
(4) 1-4 w (n= 111)
(5) 1-3 m (n= 86)
(6) 3-6 m (n= 97)
(7) 6-12 m (n= 95)
(8) 1-3 years
(9) 3-5 years
(10) 5-8 years
(11) 8-12 years
(12) 12-16 years</td>
<td>Mean QTc values (ms)(2nd percentile - 98th percentile)
<table>
<tr><td>Mean QTc (ms)</td><td>0-1d</td><td>1-3d</td><td>3-7d</td><td>1-4w</td><td>1-3m</td><td>3-6m</td><td>6-12m</td></tr>
<tr><td>F</td><td>431
(362- 488)</td><td>424
(372- 487)</td><td>430
(383 - 482)</td><td>423
(383 - 482)</td><td>444
(391 - 480)</td><td>442
(383 - 489)</td><td>434
(387 - 482)</td></tr>
<tr><td>M</td><td>429
(368 - 489)</td><td>437
(370 - 489)</td><td>430
(372 - 480)</td><td>423
(393 - 471)</td><td>438
(391 - 487)</td><td>443
(370 - 490)</td><td>436
(395 - 489)</td></tr>
</table>

Values up to 490ms may be normal in the first 6 months of life
Upper limits of normal (ULN)
F (3-6m): 489 ms
M (3-6m or 1-3y): 490 ms
Mean QTc values: ≤ 440 ms in all age groups
ULN F: 25-49 ms higher than the values accepted as normal *
ULN M: 24-46 ms higher than the values accepted as normal *

* Normal values according to Rijnbeek et al.[26] (new normal limits for the paediatric electrocardiogram)</td>
</tr>
<tr>
<td>Ulrich
(2014) [22]</td>
<td>Heart rate-corrected QT interval evolution in premature infants during the first week of life</td>
<td>114 neonates</td>
<td>3 cohorts based on gestational age
(A) 31-34 weeks (n = 30)
(B) 34-37 weeks (n = 57)
(C) > 37 weeks
(n = 27)</td>
<td>Mean QTc and Heart rate ± SD during the first week of life

Cohort A-B-C: significant decrease from birth until day 4
Cohort A-B:
Stabilization after day 4 with no significant variation
Mean QTc on day 1 in preterms (A) significantly longer than cohort B or C
Cohort C:
Daily QTc significantly shorter than cohort A from day 1-3 and shorter than B from day 2-4</td>
</tr>
<tr>
<td>Uygur
(2019) [27]</td>
<td>Normal electrocardiogram values of healthy children</td>
<td>1305 children aged 0 days - 16 years</td>
<td>10 groups based on age
1. 0-7 days (n= 89)
2. 7-30 days (n= 103)
3. 1-3 months (n= 89)
4. 3-6 months (n= 91)</td>
<td>Median QTc ms (2nd - 98th percentile)
<table>
<tr><td></td><td>0-7 days</td><td>7-30 days</td><td>1-3 months</td><td>3-6 months</td><td>6-12 months</td></tr>
<tr><td>All cases</td><td>412 (388 - 432)</td><td>411 (383 - 428)</td><td>412 (372 - 430)</td><td>414(383 - 432)</td><td>416 (386 - 432)</td></tr>
<tr><td>Females</td><td>412 (390 - 431)</td><td>411 (379 - 428)</td><td>412 (380 - 429)</td><td>417 (397 - 437)</td><td>417 (400 - 429)</td></tr>
</table></td>
</tr>
</table>

<table>
<tr>
<td></td>
<td></td>
<td></td>
<td>5. 6-12 months (n= 95)
6. 1-3 years (n= 103)
7. 3-5 years (n= 99)
8. 5-8 years (n= 164)
9. 8-12 years (n= 122)
10. 12-16 years (n= 208)</td>
<td>
<table>
<tr><td>Males</td><td>412 (379 - 436)</td><td>411 (385 - 429)</td><td>412 (372 - 431)</td><td>412 (380 - 431)</td><td>416 (381 - 435)</td></tr>
</table>
</td>
</tr>
<tr>
<td>Paerregaard (2021) [18]</td>
<td>Defining the normal QT interval in newborns: the natural history and reference values for the first 4 weeks of life</td>
<td>14164 newborns
52% M
48% F</td>
<td>/</td>
<td>Corrected QT interval in different age groups
Median values (2nd - 98th percentile)
<table>
<tr><td></td><td>all ages</td><td>0-6 days</td><td>7-13 days</td><td>14-20 days</td><td>21- 28 days</td></tr>
<tr><td>QTcbaz (ms)</td><td>419 (373- 474)</td><td>413 (364- 484)</td><td>416 (372- 466)</td><td>426 (383- 474)</td><td>432 (385- 478)</td></tr>
<tr><td>QTcHod (ms)</td><td>419 (373- 472)</td><td>406 (360- 461)</td><td>418 (347- 472)</td><td>426 (387- 476)</td><td>432 (392- 478)</td></tr>
<tr><td>QTcFri (ms)</td><td>364 (320- 414)</td><td>365 (322- 434)</td><td>360 (317- 403)</td><td>368 (322- 408)</td><td>372 (322- 415)</td></tr>
<tr><td>QTcFra (ms)</td><td>363 (327- 405)</td><td>367 (330- 425)</td><td>360 (325- 393)</td><td>365 (328- 397)</td><td>367 (328- 403)</td></tr>
</table>
</td>
</tr>
</table>

Table S8. Data extraction of 9 articles concerning covariates influencing QTc - organized by covariate.

Author (year)	Title	Patients n* (age)	Study population characteristics	Covariate	Key findings
Emmanouilides (1965) [33]	The electro-cardiogram in normal newborn infants: correlation with hemodynamic observations	28 normal newborn infants aged 1-27 hours	/	Pulmonary and systemic arterial pressure	**No correlation between**<br>• QT interval and degree of pulmonary hypertension<br>• QT interval and presence of a left-to-right shunt<br>**Average QT interval 250 ms**<br><br>Mean PAP: 22-52 mmHg<br>Mean systemic pressure: 36-68 mmHg<br>No statistical difference in PAP in babies < 13h (37.8 mmHg) ⇔ babies >13h (33.3 mmHg)
Walsh (1975) [32]	ECG changes during the first 5-6 days after birth	114 full term newborns	Groups based on moment of cord clamping after delivery of the feet<br>(1) late cord clamping (3-5 min)<br>(2) early cord clamping (<4sec)<br>(3) cord stripping	Cord clamping	QTc interval during the first week of life<br>• Early clamped infants: decreases during the first week of life<br>• Late clamped infants: decreases during the first week of life<br>• Longer QTc interval on the first day of life in infants with cord stripping
Giacoia (1978) [37]	Q-oTc interval and blood calcium levels in newborn infants	27 full term infants<br><br>77 preterm infants	(A) 27 FT<br>⟶ 11: hypoCa<br>⟶ Mean BW: 3167 ± 387 g<br><br>(B) 43 ill PT<br>⟶ Mean BW: 1565 ± 475 g<br>⟶ Mean GA: 31.1 ± 3.3 w<br><br>(C) 20 PT	Elektrolyte disturbances	ECG recordings during the first three days of life<br>Group A (27 FT)<br>• Statistically **significant** correlation between total and ionized calcium and QoTc values ($p < 0.05$)<br>• Diagnosis of hypocalcemia could not be predicted by QoTc<br>Group B (43 ill PT)<br>• **No statistical** correlation between both total and ionized calcium and QoTc values<br>Group C (20 PT)<br>• Statistically **significant** correlation between both total and ionized calcium and QoTc values<br>Group D<br>• Infusion of calcium gluconate → increase ionized calcium levels associated with shortening of QoTc in 11/12 patients

<table>
<tr>
<td></td>
<td></td>
<td></td>
<td>⟶ Mean BW: 1560 ± 582 g
⟶ Mean GA:
32 ± 2.8 w

(D) 13 hypocalcemic infants from (B), QoTc and total and ionized Ca measured before and after infusion with calcium gluconate 50mg/kg/24 h

(E) 15 normocalcemic from (A)

(F) 23 normo calcemic infants, 9 from (C), remaining 14 normal premature < 72 h of age</td>
<td></td>
<td>Differences between pre- and post-infusion values for ionized calcium and QoTc when subjected to regression analysis ⟶ significant
T = 2.55, p < 0.05
<table>
<tr><td></td><td>Before transfusion</td><td>After transfusion</td></tr>
<tr><td>Mean ionized calcium level (± SD)</td><td>243 ± 0.136 mg/dl</td><td>307 ± 0.145 mg/dl</td></tr>
<tr><td>Mean QoTc (± SD)</td><td>237 ± 13 ms</td><td>205 ± 12 ms</td></tr>
</table>
Group E (FT)
Mean total calcium (± SD) = 9.1 ± 0.17 mg/dl
Mean ionized calcium (±SD) = 3.42 ± 0.07 mg/dl
Mean QoTc = 220 ± 8 ms
Group F (PT)
Mean total calcium (± SD) = 7.79 ± .13 mg/dl
Mean ionized calcium (± SD) = 3.54 ± 0.08 mg/dl
Mean QoTc = 182 ± 10 ms</td>
</tr>
<tr>
<td>Horan
(2007) [34]</td>
<td>The effect of temperature on the QTc interval in the newborn infant receiving extracorporeal membrane oxygenation (ECMO)</td>
<td>27 neonates</td>
<td>Median GA:
40 w
(31-41 w)

Mild hypothermia during the first 5 days on ECMO

Group 1
(n = 7): 37°C for 5 days

Group 2</td>
<td>Hypothermia</td>
<td>Mean QTc ms (95th percentile) during first 24h of cooling
37°C = 431 ms (506)
36°C = 459 ms (521)
35°C = 445 ms (516)
34°C = 465 ms (531)
34°C for 48h = 466 ms (521)
QTc increased by 3.12 ms (95% CI 6.17-0.84 p = 0.04) for each degree fall in body temperature
No significant relation between QTc and rectal temperature during rewarming period

Median QT (ms) and median QTc (ms) values for study groups during cooling and rewarming
<table>
<tr><td></td><td>Group 1</td><td>Group 2</td><td>Group 3</td><td>Group 4</td><td>Group 5</td></tr>
</table></td>
</tr>
</table>

<table>
<tr><td></td><td></td><td></td><td>(n = 5): 36°C for 24h than rewarmed

Group 3
(n = 5): 35°C for 24h than rewarmed

Group 4
(n = 5): 34°C for 24h than rewarmed

Group 5
(n = 5): 34°C for 48h than rewarmed</td><td></td><td>
<table>
<tr><td>QT for first 24h on ECMO</td><td>289
(248-439)</td><td>326
(263-395)</td><td>335
(260-425)</td><td>328
(208-444)</td><td>349
(273-408)</td></tr>
<tr><td>QTc for the first 24h</td><td>430
(360-530)</td><td>454
(400-536)</td><td>439
(355-534)</td><td>478
(315-539)</td><td>465
(385-534)</td></tr>
<tr><td>QT for the first 48h</td><td>Not cooled</td><td>Not cooled</td><td>not cooled</td><td>not cooled</td><td>344
(363-411)</td></tr>
<tr><td>QTc for the frist 48h</td><td>Not cooled</td><td>Not cooled</td><td>Not cooled</td><td>Not cooled</td><td>466
(380-539)</td></tr>
<tr><td>QTc during rewarming</td><td>428
(367-519)</td><td>458
(397-527)</td><td>425
(351-525)</td><td>456
(379-529)</td><td>467
(380-539)</td></tr>
<tr><td>QTc when rewarmed to 37°C</td><td>448
(351-530)</td><td>438
(391-516)</td><td>419
(343-486)</td><td>441
(383-545)</td><td>442
(377-518)</td></tr>
</table>
</td></tr>
<tr><td>Shabestari (2019) [36]</td><td>Electrocardiographic corrected QT (QTc) dispersion value as a predictor for estimation of neonatal mortality in pre-term neonates</td><td>127 neonates</td><td>Population divided in 4 groups

(1): FT > 37 w
(A): healthy
(B): suffering illnesses or signs and symptoms

(2) PT < 37 w
(A): healthy
(B): suffering illnesses or signs and symptoms</td><td>Illness</td><td>ECG recordings on postnatal day 3, 7 and 28 day

• Normal full term neonates: significantly higher QT values compared to ill full term neonates
• Ill preterm neonates: significantly higher QTc values compared to normal preterm neonates

• No mortality in normal or ill full term neonates and normal preterm neonates
• Ill preterm neonates
⟶ 8 died in the first 3 days of life
⟶ Retrospectively QTd and QTcd significantly higher in dead ill preterm than alive preterm neonates</td></tr>
<tr><td>Haddad (1979) [38]</td><td>Effect of sleep state on the</td><td>12 full term newborns</td><td>Birth weight: 2900 - 4560 g</td><td>Sleep</td><td>ECG recordings at 2 weeks, 1 month, 2 months, 3 months and 4 months of age
1. REM-sleep</td></tr>
</table>

<table>
<tr>
<td></td>
<td>QT interval in normal infants</td>
<td>(7 males - 5 females)</td>
<td>GA: 38 - 42 w</td>
<td></td>
<td>QTc intervall: 0.384-0.466 ms
mean QTc = 0.433 ms, SD 0.017 ms
2. non-REM (quiet)
QTc interval: 0.391-0.479 ms
mean QTc = 0.439 ms, SD 0.019 ms
QTc interval longer in non-REM than in REM sleep
⟶ Significant difference in REM and non-REM sleep at all ages</td>
</tr>
<tr>
<td>Benatar (2002) [41]</td>
<td>QT interval in normal infants during sleep with concurrent evaluation of QT correction formulae</td>
<td>130 full term newborns aged 4-72 weeks</td>
<td>Birth weight: 2480 - 4300 g</td>
<td>Sleep</td>
<td>24 ECG recordings per infant. 3 per hour during 8- hour monitoring
Mean QT ± SD (ms): 288 ± 21.7
Mean QTc ± SD (ms)
Bazett: 416 ± 23.4
95%: QTc < 450 ms
98%: QTc < 480 ms
98% percentile: QTc = 464 ms
Hodges: 405 ± 21.3
Fridericia: 369 ± 20.4
Framingham: 368 ± 16.7</td>
</tr>
<tr>
<td>Ariagno (2003) [39]</td>
<td>Effect of position on sleep, heart rate variability and QT interval in preterm infants at 1 and 3 months' corrected age</td>
<td>16 asymptomatic preterm infants</td>
<td>6 females
10 males
Mean GA: 34.4 ± 2.2 weeks (28-36w)

Mean BW: 2326 ± 439 g (1172 – 3000 g)</td>
<td>Sleep</td>
<td>ECG recordings at one and three months corrected age
<table>
<tr><td>QS (quiet sleep)</td><td>Prone Mean ± SD</td><td>Supine Mean ± SD</td><td>P value</td></tr>
<tr><td>Average QTc, 1m</td><td>451 ± 23ms</td><td>443 ± 25ms</td><td>0.03</td></tr>
<tr><td>Average QTc, 3m</td><td>418 ± 19ms</td><td>422 ± 18ms</td><td>NS</td></tr>
<tr><td>AS (Active sleep)</td><td>Prone Mean ± SD</td><td>Supine Mean ± SD</td><td>P value</td></tr>
<tr><td>Average QTc, 1m</td><td>444 ± 30ms</td><td>440 ± 23ms</td><td>NS</td></tr>
<tr><td>Average QTc, 3m</td><td>417 ± 21ms</td><td>416 ± 21ms</td><td>NS</td></tr>
</table>
One month old
QTc: significantly longer in prone position
% active sleep (AS) significantly lower in supine position
Incidence of sleep transition and % indeterminate sleep (IS): significantly higher in supine position
No difference in quiet sleep (QS) and total sleep</td>
</tr>
</table>

					**Three months old**<br>• **No significant difference between prone and supine**<br>• Prone may increase duration of QS right after feeding in preterm infants
Krasemann (2010) [40]	The corrected QT interval in 24h ECGs in neonates	100 neonates aged 2- 11 days<br>51 M<br>49 F	Mean weight: 3390 g<br>GA: 35- 41 weeks (mean 40 weeks)	Sleep	Ambulantory 24 hour ECG recordings to compare QTc during sleep vs awake periods<br>• No significant differences between the sexes in any period<br>• Significant difference of QTc in lead II between sleeping and awake periods in both genders and in the entire cohort

Table S9. Data extraction of 23 articles concerning medicines influencing QTc – organized by medicine

<table>
<tr><th>Author (Year)</th><th>Patients n* (age)</th><th>Study population characteristics</th><th>Type of medication</th><th>Dose of medication</th><th>QT or QTc-interval
Key findings</th></tr>
<tr><td>Bernardini (1997) [44]</td><td>49 infants aged 1-43 days</td><td>Mean BW:
2240 g
(820 - 4200 g)

Mean GA:
34.6w
(25-41 w)</td><td>Cisapride</td><td>Mean 0.84 mg/kg/day
(0.42 - 1.6)</td><td>ECG recordings at baseline and 2-6 days after administration
QTc intervals are prolonged if > 450 ms
Significant prolongation of QTc after starting cisapride (p = 0.0001)

QTc before and after treatment with cisapride
<table>
<tr><th></th><th>Before</th><th>After</th></tr>
<tr><td>Mean QTc (ms)
Range (ms)</td><td>395
356-446</td><td>418
371-504</td></tr>
</table></td></tr>
<tr><td>Khogphattha-nayothin (1998) [45]</td><td>101 infants</td><td>Divided into:
(1) Cisapride treatment started at inclusion time
(2) Already receiving cisapride for at least 2 days</td><td>Cisapride</td><td>Mean 0.81 ± 14 mg/kg/day</td><td>Significant prolongation of QTc

Group 1
ECG recordings at baseline, 1-2h after administration and 2-7 days after administration
Mean values ± SD
<table>
<tr><th></th><th>HR (bpm)</th><th>QTc (ms)</th></tr>
<tr><td>Before</td><td>142.2 ± 25.2</td><td>393.9 ± 27</td></tr>
<tr><td>After</td><td>139.4 ± 19.7</td><td>409.4 ± 30</td></tr>
<tr><td>Change</td><td>-2.9 ± 23.1</td><td>+ 15.5 ± 25</td></tr>
<tr><td>P value</td><td>0.50</td><td>0.002</td></tr>
</table>
* QTc significant longer after cisapride

Group 2
First 40 patients: 12 lead ECG recording on peak dose level cisapride
Other patients: random ECG recording
13 patients: QTc > 440 ms
(11/13 other risk factors)</td></tr>
</table>

<table>
<tr>
<td>Costalos
(2000) [42]</td>
<td>20 infants
aged 14 days
12 M
8 F</td>
<td>Mean GA: 30.5 w
Mean BW: 1320 g

Divided into
(1) Cisapride (n = 10)
(2) Placebo
(n = 10)</td>
<td>Cisapride</td>
<td>0.3 mg/kg/day</td>
<td>ECG recording at baseline and 7 days after administration
QTc values are prolonged if > 450 ms

Mean QTc values (ms) in infants treated with cisapride versus placebo
<table>
<tr><td></td><td>Cisapride</td><td>Placebo</td></tr>
<tr><td>Mean QTc (ms)
Range (ms)</td><td>365
300-420</td><td>393
260-460</td></tr>
</table>
Significant shortening of QTc in cisapride-group</td>
</tr>
<tr>
<td>Ramirez-
Mayans
(2000) [43]</td>
<td>120 full term
infants aged
1 months to
18 years</td>
<td>Divided into:
(1) Already taking cisapride for GI-reflux (n = 63)
(2) Placebo
(n = 57)</td>
<td>Cisapride</td>
<td>0.6 mg/kg/day</td>
<td>ECG recording at baseline and 15 days after administration only if prolonged QTc
QTc values are prolonged if > 460 ms

Mean QTc values ± SD (ms) in infants treated with cisapride versus placebo
<table>
<tr><td>Mean QTc in age:</td><td>Cisaprid
e</td><td>Placebo</td></tr>
<tr><td>< 4m
p = 0.004</td><td>410 ± 27</td><td>428 ± 23</td></tr>
<tr><td>5-12m</td><td>412 ± 38</td><td>411 ± 38</td></tr>
</table>
Significant shortening of QTc in age group < 4 months in cisapride-group

Group 1: 5/63 prolonged
Group 2: 6/57 prolonged</td>
</tr>
<tr>
<td>Vandenplas
(2000) [55]</td>
<td>227 infants
(1) cisapride
(n: 150)
(2) controls
(n: 127)</td>
<td>Divided into:
(1) < 3 months
(2) between 3-6 months
(3) > 6 onths</td>
<td>Cisapride</td>
<td>1. Mean 0.80 mg/kg/day (0.35-1.55)

2. Mean 0.80 mg/kg/day (0.23-1.38)

3. Mean 0.72 mg/kg/day (0.32-1.41)</td>
<td>Statistically significant increase in QTc in infants < 3 months of age receiving cisapride (p <0.001)

Infants < 3 months:
• Mean QTc (cisapride): 500ms
• Mean QTc (controls): 447 ms</td>
</tr>
</table>

<table>
<tr>
<td>Benatar
(2001) [53]</td>
<td>211 infants aged 1 week to 13.5 months</td>
<td>(1) Cisapride (n = 86)
(2) Controls
(n = 127)

Divided into 2 groups
1. < 3 m
2. > 3 m</td>
<td>Cisapride</td>
<td>Mean 0.81 mg/kg/day</td>
<td>Continuous ECG recordings during polysomnography
12 lead ECG recording in cisapride treated infants

Mean ECG parameters ± SD (ms)
<table>
<tr><td></td><td>Group 1 <3 m</td><td>Group 2 >3 m</td></tr>
<tr><td>HR (12 lead)</td><td>147 ± 16</td><td>141 ± 16.7</td></tr>
<tr><td>HR (8h)
cisapride
control</td><td>128 ± 10.5
133 ± 10</td><td>120.5 ± 9.5
120 ± 12</td></tr>
<tr><td>QT (12 lead)</td><td>276 ± 23</td><td>274 ± 21</td></tr>
<tr><td>QT (8h)
cisapride
control</td><td>308.8 ± 20.1*
283.7 ± 16.2</td><td>298 ± 14.3
293 ± 18.9</td></tr>
<tr><td>QTc (12 lead)</td><td>432.4 ± 28.4***</td><td>419 ± 23.6</td></tr>
<tr><td>QTc (8h)
cisapride
Control</td><td>448 ± 25.5**
419.5 ± 18.6</td><td>417.9 ± 21.64
412 ± 20</td></tr>
</table>
* p <0.001 compared with control
** p < 0.001 compared with controls and older than 3 months of age
*** p <0.001 compared with younger than 3 months of age

Significant prolongation in infants younger than 3 months treated with cisapride
No significant difference in QTc between term and preterm infants
Significant relation between cisapride plasma levels and age</td>
</tr>
<tr>
<td>Cools
(2001) [52]</td>
<td>10 preterm infants</td>
<td>- Mean GA 36.6 w (26.6-33.4 w)
- Mean BW 1448 g (1100-1880 g)</td>
<td>Cisapride</td>
<td>Mean 0.8mg/kg/day</td>
<td>ECG recording at baseline and 72 hours after administration

Mean QTc values before cisapride treatment and after 72hours (Mean QTc ± SD ms)
<table>
<tr><td></td><td>Baseline</td><td>After 72h</td><td>P value</td></tr>
<tr><td>Mean QTc</td><td>423 ± 20</td><td>461 ± 20</td><td>0.0007</td></tr>
</table></td>
</tr>
</table>

<table>
<tr><td></td><td></td><td></td><td></td><td></td><td>Significant prolongation of the QTc interval during treatment with cisapride

Prolongation of QTc value not related to GA, rather to postnatal age
⇒ not prematurity but young age is an important factor in the effect of Cisapride on the QTc interval</td></tr>
<tr><td>Dubin
(2001) [50]</td><td>25 preterm infants with mean 35 weeks PCA
(15/10 M/F)</td><td>Mean GA 29 ± 3 weeks

Mean BW 1190g ± 400 g

Divided into:
1. GA < 31w
(n = 18)
2. GA > 31w
(n = 7)</td><td>Cisapride</td><td>0.4 mg/kg/day
Maximal 0.8 mg/kg/day</td><td>ECG recording at baseline and after 5 t1/2. If dosage increases, again after 5 t1/2
QTc values are prolonged if > 450 ms

Age in weeks, mean values (ms) ± SD
<table>
<tr><th>GA</th><th>PCA</th><th>QTc before start</th><th>QTc after start</th></tr>
<tr><td>< 32</td><td>35 ± 3</td><td>410 ± 0.02</td><td>440 ± 0.02*°</td></tr>
<tr><td>≥ 32</td><td>38 ± 3</td><td>410 ± 0.02</td><td>420 ± 0.02</td></tr>
</table>
* p ≤ 0.05 comparing < 32 w GA with ≥ 32 w
° p ≤ 0.05 comparing before and after treatment
8 infants with QTc > 450 ms
Dose:
- 13 infants 0.4 mg: 7/13 prolonged
- 12 infants 0.8 mg: 5/13 prolonged

Significant prolongation of QTc in group 1</td></tr>
<tr><td>Semama
(2001) [46]</td><td>21 term infants</td><td>Mean BW 3065 ± 579 g
(1700-4180 g)

Mean GA 39.3 ± 1.4 w
(37- 41 w)</td><td>Cisapride</td><td>Mean 0.19 mg/kg, 4x/day
(range: 0.18-0.21)</td><td>ECG recording at baseline, at 48 h after start treatment, at 7 days after start treatment and at 15 days after start treatment

Mean values (ms) ± SD
Range
<table>
<tr><th>Baseline</th><th>After 48h</th><th>After 7d</th><th>After 15d</th></tr>
<tr><td>397 ± 21
(335- 423 ms)</td><td>418 ± 28
(386- 461 ms)</td><td>431± 23
(386-461 ms)</td><td>447± 38
(414 - 515 ms)</td></tr>
</table>
Significant prolongation in QTc when treated with cisapride (p <0.01) (mean QTc values)</td></tr>
</table>

<table>
<tr>
<td>Zamora
(2001) [47]</td>
<td>11 preterm infants and 24 term infants aged 8 to 221 days</td>
<td>GA 25-35 w</td>
<td>Cisapride</td>
<td>1 mg/kg/day</td>
<td>ECG recording at baseline and 3 days after administration
QTc values are prolonged if > 450 ms

Mean values (ms) ± SD
<table>
<tr><td>Term - before</td><td>Term - after</td><td>Preterm - before</td><td>Preterm - after</td></tr>
<tr><td>395.5 ± 20.4</td><td>417 ± 31.3</td><td>399 ± 33.3</td><td>416.4 ± 43.9</td></tr>
</table>
5/35 infants with QTc > 450 ms
- 2/11 preterm infants
- 3/11 term infants
Significant prolongation of QTc after starting cisapride in both groups</td>
</tr>
<tr>
<td>Benatar
(2002) [54]</td>
<td>15 preterm infants</td>
<td>Mean GA 30.5 weeks (26.5-33.5 w)

Mean postnatal age: 24 days
(5- 51 days)</td>
<td>Cisapride</td>
<td>0.8mg/kg/ day (0.76-0.89)</td>
<td>ECG recording at baseline and 3 days after administration
Mean values (ms) ± SD
<table>
<tr><td></td><td>Before</td><td>On cisapride</td></tr>
<tr><td>QT ±SD
Range</td><td>272 ± 28
236-326</td><td>294 ± 32*
250 - 348</td></tr>
<tr><td>QTc ± SD
Range</td><td>429 ± 29
386 - 492</td><td>454 ± 29**
422 - 526</td></tr>
</table>
* Significant longer (p <0.03)
** Significant longer (p <0.02)
Significant prolongation of QT and QTc interval during treatment with cisapride</td>
</tr>
<tr>
<td>Chhina
(2002) [49]</td>
<td>44 preterm infants and 6 term infants</td>
<td>Mean GA 29.9 weeks
Mean PCA 34.5 weeks</td>
<td>Cisapride</td>
<td>0.8mg/kg/ day</td>
<td>ECG recording at basline and 3, 5, 7 and 14 days after administration
QTc values are prolonged if > 450ms

Prolongation of QTc in 15/50 infants (30%) after starting cisapride
No correlation with GA and PCA
13/15 had normalization of QTc interval after 14 days</td>
</tr>
<tr>
<td>Cools
(2003) [51]</td>
<td>31 preterm infants</td>
<td>Divided into:
(1) Cisapride (n =16)
(2) Cisapride (n = 15)</td>
<td>Cisapride</td>
<td>0.2mg/kg, 6 hourly

0.1mg/kg, 3 hourly</td>
<td>ECG recording at baseline and 72 hours after administration
Mean QTc ± SD (ms)
<table>
<tr><td></td><td>Before</td><td>After 27h</td><td>P value</td></tr>
<tr><td>Group A</td><td>422 ± 20</td><td>460 ± 23</td><td>0.0006</td></tr>
</table></td>
</tr>
</table>

<table>
<tr>
<td></td>
<td></td>
<td></td>
<td></td>
<td></td>
<td>
<table>
<tr><td>Group B</td><td>396 ± 22</td><td>434 ± 27</td><td>0.0003</td></tr>
</table>

Mean QTc both before and after 72h longer in group A than B (p = 0.002 and p = 0.01 respectively)

Linear regression: significant positive correlation between the change of QTc interval and serum cisapride concentration

Significant prolongation of QTc after 72 hours of treatment in both groups
</td>
</tr>
<tr>
<td>Corvaglia (2004) [56]</td>
<td>Cisapride group:
46 preterm infants (21 M, 25 F)
→ 31 AGA
→ 15 SGA

Control group:
50 preterm infants (22M, 28 F)
→ 35 AGA
→ 15 SGA</td>
<td>Cisapride group:
- Mean BW 1350 g (410-2800 g)
- Mean PCA 31 w (25-35 weeks)
- Median age at beginning of treatment 10 d
(7-12 d)

Median length of treatment: 10 d (5- 25 d)

Control group:
- Mean BW 1300 g (510-2350 g)
- Mean PCA 30.5 w</td>
<td>Cisapride</td>
<td>16 infants: 0.1 mg/kg, 8 hourly (0.3mg/kg/d)

30 infants: 0.2mg/kg, 6 hourly (0.6mg/kg/d)</td>
<td>
2 ECG recordings with a mean interval of five days

Mean QTc interval ± SD (ms)
<table>
<tr><td></td><td colspan="2">Treated</td><td colspan="2">Controls</td></tr>
<tr><td></td><td>Pre</td><td>Post</td><td>1st ECG</td><td>2nd ECG</td></tr>
<tr><td>Total</td><td>390 ± 10</td><td>400 ± 20</td><td>387± 15</td><td>382 ± 19</td></tr>
<tr><td>SGA</td><td>397 ± 16</td><td>416 ± 34</td><td>394 ± 16</td><td>388 ± 20</td></tr>
<tr><td>AGA</td><td>386 ± 15</td><td>396 ± 16</td><td>384 ± 14</td><td>379 ± 18</td></tr>
</table>

→ whole population: p = 0.0001
→ AGA: p = 0.0001
→ AGA: p = 0.01
Significant prolongation in QTc during treatment than in controls

SGA preterms higher baseline QTc values than AGA preterms and have greater mean QTc lengthening during treatment (21 ms SGA ⇔ 9 ms AGA) with significant difference (p = 0.041)

Longer QTc intervals in SGA than AGA preterm infants and higher susceptibility to QTc lengthening during cisapride therapy
</td>
</tr>
</table>

<table>
<tr><td>Zamora
(2004) [48]</td><td>14 term and
17 preterm
infants</td><td>- GA 28- 36 w
- Median age 29 d (3-132 d)</td><td>Cisapride</td><td>0.8 mg/kg/day</td><td>ECG recording at baseline and 3 days after administration
Significant prolongation after start cisapride only in preterm infants

ECG in term and preterm infants before and after cisapride treatment (mean ± SD)<table><tr><td></td><td colspan="2">Preterm (n = 17)</td><td colspan="2">Term (n = 14)</td></tr><tr><td></td><td>Pre</td><td>Post</td><td>Pre</td><td>Post</td></tr><tr><td>PR ms</td><td>92 ± 2</td><td>92 ± 1</td><td>100 ± 3</td><td>98 ± 3</td></tr><tr><td>QTc ms</td><td>408 ± 7</td><td>433 ± 7*</td><td>400 ± 11</td><td>410 ± 6</td></tr><tr><td>QTd ms</td><td>31 ± 1</td><td>27 ± 3</td><td>30 ± 3</td><td>31 ± 2</td></tr></table>* p < 0.05 before versus after cisapride in preterm infants</td></tr>
<tr><td>Kohl
(2005) [57]</td><td>59 infants</td><td>Divided into:
(1) Cisapride (n =29)

GA 29 w
(23.1 - 31.7 w)

BW in
- ELBW 812 g
- LBW 1490 g

(2) Placebo (n = 30)
GA: 30.4 w
(24.7- 31.9)

BW in
- ELBW 806 g
- LBW 1450 g</td><td>Cisapride</td><td>0.8 mg/kg/day</td><td>ECG recording at baseline and 3 days after administration

Mean values after three days (ms)<table><tr><td colspan="2">ELBW</td><td colspan="2">LBW</td></tr><tr><td>cisapride</td><td>placebo</td><td>cisapride</td><td>placebo</td></tr><tr><td>420 (360- 450)</td><td>380 (360 - 400)</td><td>420 (350 - 450)</td><td>380 (340- 430)</td></tr><tr><td colspan="2">p = 0.026</td><td colspan="2">p = 0.052</td></tr></table>Significant prolongation of QTc after starting cisapride in ELBW group

One infant experienced paroxysmal supraventricular tachycardia</td></tr>
<tr><td>Berul
(2006) [58]</td><td>36 neonates</td><td>PCA 28- 54 w

Divided based on PCA
- 28-36 w
- 37-42 w</td><td>Cisapride</td><td>Day 1
- Single oral dose of 200 μg/kg</td><td>ECG recordings
1. 12 lead ECG for screening
2. 6 lead ECG at
→ baseline
→ day 1: 0.5, 1, 2, 4, 8, 12, 24h</td></tr>
</table>

		- 43-54 w		- 200 **μ**g/kg every 6h	→ *day 4: 1 and 6 h after 12th dose*<br>→ *day 7: 1 and 2 h after 24th dose*<br>3. *Additional 12 lead ECG on*<br>→ *day 4: 2h after 12th dose*<br>→ *day 7: 6h after 24th dose*<br>**Uncorrected QT interval** changed significantly from baseline on day 2-7 only in PCA group 28-36w<br><br>QTc significantly **increased** at various time points on day 1<br>On day 2-7 further statistically significant increases in QTc<br><br>Baseline<br>• Mean QT ± SD<br>o 28-36 w: 238 ± 6.5 ms<br>o 37-42 w: 265 ± 6 ms<br>o 43-54 w: 260 ± 11 ms<br>• Mean QTc ± SD<br>o Bazett: 396 ± 4.3 ms<br>o Frederica: 340 ± 4.3 ms<br>o Study specific: 252 ± 2.6 ms<br>Highest values for mean change in QTcBaz and QTcFri<br>• Day 1: 7.5 ± 3 ms at 4h post dose<br>• Day 1: 6.3 ± 2.9 ms at 2h post dose<br>Highest mean<br>• QTcBaz = 417 ± 7.9 ms on day 7; 1.45h post dose<br>• QTcFri = 359 ± 7.7 ms on day 7; 1.45 post dose<br><br>Changes in QT and QTc fall within the spectrum of expected with great variability between individuals
Djeddi (2008) [59]	31 neonates	Divided into 3 groups<br>(1) GA > 37 w<br>(2) GA between 32 - 37 w<br>(3) GA < 32 w	Domperidone	Mean 1.13 ± 0.07 mg/kg/day	*3 ECG recordings*<br>1. *Baseline*<br>2. *2.5 ± 1.5 days after start treatment*<br>3. *48 hours after treatment stop in patient with QTc > 450 ms during treatment*<br>*QTc values are prolonged if > 450 ms*<br>*Dompiridone administration stopped if QTc > 450 ms during treatment*<br><br>Significant **prolongation** of QTc interval during treatment p <0.01<br><br>Mean variation: 14 ms with mean duration of 60 hours

<table>
<tr><td></td><td></td><td></td><td></td><td></td><td>
<table>
<tr><td>Baseline</td><td>During treatment</td></tr>
<tr><td>373 ± 4.87 ms</td><td>387.2 ± 5.1 ms</td></tr>
</table>
Significant differences in QTc between GA groups ($p < 0.05$)
<table>
<tr><td>Group 1</td><td>Group 2</td><td>Group 3</td></tr>
<tr><td>364.8 ± 6.8 ms</td><td>398 ± 9.3 ms</td><td>377.9 ± 7.4 ms</td></tr>
</table>
In 48.4% prolongation of QTc interval with 12 ms

Oral domperidone has a significant impact on QTc interval in infants aged 32 to >37 weeks not in infants < 32 weeks
</td></tr>
<tr><td>Günlemez
(2010) [60]</td><td>40
premature
infants
26 M
14 F
with mean
age 32 days</td><td>GA: 28.8 w
(24-33 w)</td><td>Domperidone</td><td>1 mg/kg/day</td><td>
ECG recording at baseline, 3, 5 and 7 days after administration
QTc values are prolonged if > 450 ms

No significant prolongation after start domperidone
<table>
<tr><td>Baseline QTc</td><td>370 ms (0.03)</td></tr>
<tr><td>QTc at day 3</td><td>380 ms (0.03)</td></tr>
<tr><td>QTc at day 7</td><td>370 ms (0.04)</td></tr>
<tr><td>QTc at day 14</td><td>370 ms (0.03)</td></tr>
</table>
2/7 infants with QTc > 450 ms that returned to normal after discontinuation
</td></tr>
<tr><td>Vieira
(2012) [61]</td><td>45 infants
27 M
18 F</td><td>Mean GA 38.6w
(35.5 - 42 w)

Mean age at start of treatment 75.3d
(19-218 d)</td><td>Domperidone</td><td>0.5-1.0 mg/kg/
dose, 3-4 times
per day</td><td>
ECG recording at baseline and 1 h after intake at day 7-14

As group no statistical difference in QTc between baseline and second ECG
390 ± 20 ms ⇔ 397 ± 21 ms ($p = 0.130$)

No difference in QTc change in females
($p = 0.622$)
</td></tr>
</table>

<table>
<tr><td></td><td></td><td></td><td></td><td></td><td>Almost significant QTc change in males
(p = 0.051)

The younger the infant the larger the change in QTc, but not statistically significant</td></tr>
<tr><td>Maillard
(2001) [63]</td><td>40 infants
25 M
15 F
with mean
age 26 days</td><td>- Mean GA 25- 34 w
- Mean BW 590 - 2220 g</td><td>Doxapram</td><td>IV: 0.5 - 1
mg/kg/h

Switched to PO
in 14/40: 30
mg/kg/day</td><td>ECG recording at baseline and during the first 3 days of treatment
QTc values are prolonged if > 440 ms

Significant prolongation after start doxapram

Mean values ± SD<table><tr><td>Before doxapram</td><td>At 72h after start doxapram</td></tr><tr><td>394 ± 4 ms</td><td>409 ± 4 ms
p = 0.0065</td></tr></table>6/40 infants with QTc > 440ms</td></tr>
<tr><td>Miyata
(2007) [62]</td><td>15 infants</td><td>Mean GA 30.1 ± 2.2 w
Mean BW 1312 ± 285 g</td><td>Doxapram</td><td>0.2 mg/kg/h</td><td>ECG recording at baseline and 24 h after administration
Mean ECG parameters before and after therapy (mean ± SD)<table><tr><td></td><td>Before</td><td>After</td><td>p value</td></tr><tr><td>RR (ms)</td><td>432 ± 46</td><td>437 ± 46</td><td>< 0.05</td></tr><tr><td>PR (ms)</td><td>96 ± 15</td><td>92 ± 16</td><td>< 0.05</td></tr><tr><td>QT (ms)</td><td>368 ± 41</td><td>275 ± 7</td><td>< 0.05</td></tr><tr><td>QTc (ms)</td><td>408 ± 48</td><td>418 ± 30</td><td>< 0.05</td></tr></table>Significant changes were observed, but all values within physiological ranges</td></tr>
</table>
